# The methanolic extract of *Byrsonima intermedia* mitigates colonic injury in experimental colitis through redox restoration, cytokine modulation, and tissue repair

**DOI:** 10.1007/s10787-026-02312-4

**Published:** 2026-07-14

**Authors:** Karla Stella Figueiredo Romano, Felipe Leonardo Fagundes, Quélita Cristina Pereira, Maycon Tavares Emílio-Silva, Larissa Lucena Périco, Vinicius Peixoto Rodrigues, Gabriela Bueno, Mateus Magami Yoshitani, Giovana Pacciulli Pereira, José Aires Pereira, Carlos Augusto Real Martinez, Alessandra Gambero, Clélia Akiko Hiruma-Lima, Raquel de Cássia dos Santos

**Affiliations:** 1 Laboratory of Natural Products, Post Graduate Program in Health Sciences, São Francisco University, Bragança Paulista, São Paulo 12900-560 Brazil; 2 Department of Physiology, Institute of Bioscience, São Paulo State University, Botucatu, São Paulo 18618-689 Brazil; 3 Snyder Institute for Chronic Diseases, Cumming School of Medicine, University of Calgary, 3330 Hospital Drive NW, Calgary, AB T2N 4N1 Canada; 4 Laboratory of Medical Investigation, São Francisco Medical School, Post Graduate Program in Health Sciences, Bragança Paulista, São Paulo 12900-560 Brazil; 5 Life Science School, Pontifical Catholic University of Campinas, Campinas, 13087-571 Brazil; 6 Faculty of Pharmaceutical Sciences, State University of Campinas, Campinas, São Paulo 13081-970 Brazil

**Keywords:** TNBS-induced colitis, *Byrsonima intermedia*, Redox homeostasis, Cytokine modulation, Anti-inflammatory activity

## Abstract

This study evaluated the anti-inflammatory and antioxidant effects of a methanolic extract of *Byrsonima intermedia* leaves (EBi) in a rat model of 2,4,6-trinitrobenzene sulfonic acid (TNBS)-induced colitis. Male Wistar rats received intracolonic TNBS for lesion induction and relapse, followed by oral treatment with EBi for seven days. EBi markedly reduced the severity of colonic injury, as demonstrated by a 63% decrease in macroscopic damage, along with reductions in inflammatory infiltrate (32%) and neutrophil accumulation (52%) compared to the vehicle group. These protective effects were accompanied by significant attenuation of myeloperoxidase (MPO) activity and lipid peroxidation, together with increased superoxide dismutase (SOD) activity and glutathione levels (GSH), indicating restoration of redox homeostasis. EBi also modulated key inflammatory mediators, promoting IL-10 upregulation while downregulating IL-1*β*, MCP-1, and TLR4 expression in colonic tissue. Histological analysis confirmed substantial improvement in tissue architecture and inflammatory status. Collectively, these findings demonstrate that *B. intermedia* exerts multi-target anti-inflammatory and antioxidant actions that contribute to tissue repair in experimental colitis, supporting its potential as a promising natural therapeutic candidate for inflammatory bowel disease.

**Graphical abstract:**

Multi-target mechanisms underlying the protective effects of *Byrsonima intermedia* in experimental TNBS-induced colitis. The extract reduces macroscopic lesions, restores redox homeostasis (↑SOD, ↑GSH, ↓MDA, ↓ROS), and modulates key inflammatory mediators (↓IL-1*β*, ↓MPO, ↓MCP-1, ↑IL-10), collectively promoting tissue repair and attenuation of intestinal inflammation**.** Created in BioRender. Pereira, Q. (2025)

**Figure:**
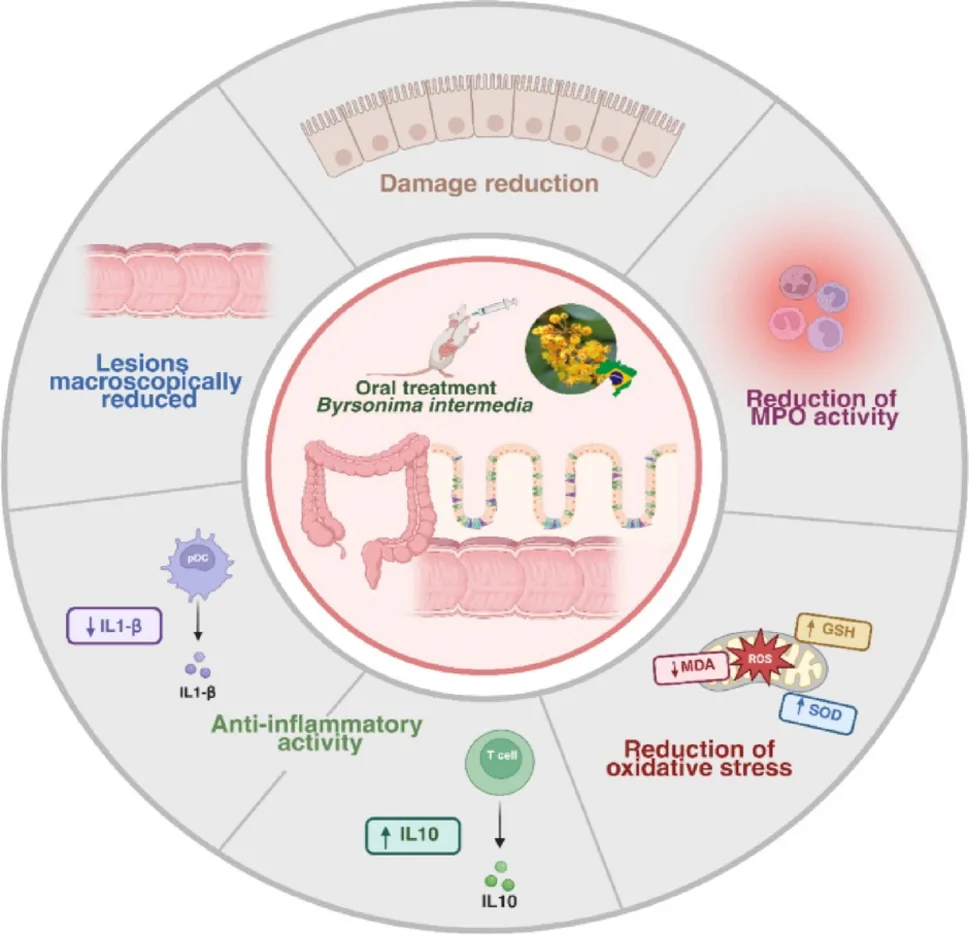

**Supplementary Information:**

The online version contains supplementary material available at https://doi.org/10.1007/s10787-026-02312-4.

## Introduction

Inflammatory bowel disease (IBD) is a life-threatening condition that occurs in multiple forms, including Ulcerative Colitis (UC) and Crohn’s disease (CD). Both diseases have chronic and relapsing causes, affecting approximately five million people worldwide (Berre et al. 2023). The main symptoms are weight loss, diarrhea, and abdominal pain. The etiology is unknown; however, scientists and physicians support an interplay between the genetic factors of the host, environment, immune system, and microbiome (Kotla and Rochev 2023). The intestinal walls become inflamed, which can persist or recur (Iliev et al. 2025). Furthermore, extraintestinal manifestations associated with IBD can occur, indicating that the disease effects extend beyond the intestinal tract and can be systemic (Rogler et al. 2021; Gupta and Choi 2025).

There is no cure for IBD, and its treatment is based on symptom control. Aminosalicylates, corticosteroids, and biological agents are usually used in IBD management, but they have severe adverse effects that reduce the quality of life without avoiding disease relapse (Villablanca et al. 2022).

*Byrsonima intermedia* A. Juss is an herb in the Brazilian Cerrado, popularly known as 'murici-pequeno,’ a shrub species found in several regions of Brazil (Anderson 1977; Nogueira et al. 2004). Plants and fruits are used to treat fever and gastrointestinal disorders, such as stomachache, diarrhea, dysentery, diuretics, and asthma (Sannomiya et al. 2007a; Lopez et al. 2001; Pinto and Bertolucci 2002). Experimentally, this species has several beneficial effects, such as anti-inflammatory and antinociceptive activities (Moreira et al. 2011; Orlandi et al. 2011), protection and repair of peptic ulcers, antimicrobial and antidiarrheal effects (Santos et al. 2012), and mutagenic activity (Sannomiya et al. 2007a). Analysis of the methanol extract of the leaves revealed that the species contained ( +)-catechin, (−)-epicatechin (Moreira et al. 2011), quercetin-3-O-beta-d-galactopyranoside, methyl gallate, gallic acid, quercetin-3-O-alpha-l-arabinopyranoside, amentoflavone, quercetin, quercetin-3-O-(2-O-galloyl)-beta-galactopyranoside, and quercetin-3-O-(2-O-galloyl)-alpha rabinopyranoside (de Cassia Dos Santos et al. 2019; Sannomiya et al. 2007b). EBi has different pharmacological actions, such as gastroprotective, antimicrobial, and healing activities (Santos et al. 2012; de Cassia Dos Santos et al. 2019). Leaves and roots are the main sources of the compounds responsible for these effects. This study aimed to evaluate the pharmacological potential of a methanolic extract of *Byrsonima intermedia* leaves (EBi) in experimental IBD induced by intracolonic administration of 2,4,6 trinitrobenzene sulfonic acid (TNBS) in male Wistar rats. Among the various experimental designs, the induction of colitis by TBNS is a widely used animal model that shares significant properties with Crohn disease in humans, allowing the study of immunopathogenesis and potential treatments (Antoniou et al. 2016).

## Material & method

### Animals

Male Wistar rats (200–220 g, 8–10 weeks old, n = 80) were provided by the Multidisciplinary Center of Biological Investigation in the Area of Animal Science (São Paulo State University, UNICAMP, Brazil). The rats were maintained at a room temperature of 22 ± 2 °C and a light/dark cycle of 12 h. The animals were fed rodent food (Presence®) and provided water ad libitum. Prior to the experiments, the animals were fasted for 12 h. All experiments were conducted after obtaining ethical approval (01.0226.2014 and no 9793170320) from the Committee of Animal Use of São Francisco University and Ethics Committee on Animal Use of the São Paulo State University and carried out under Brazilian law in the management of laboratory animals (Lei Arouca) and the ARRIVE Guidelines from NC3R.

### Drugs and reagents

Trinitrobenzene sulfonic acid (TNBS), ortho-dianisidine dihydrochloride, basic monobasic potassium phosphate, bibasic sodium phosphate, reduced L-glutathione, 2-Amino-2-(hydroxymethyl)-1,3-propanediol (TRIS), xanthine oxidase from a microbial source, formaldehyde, Triton X-100, hydrochloric acid, sodium pyrophosphate, sodium fluoride, sodium orthovanadate, catalase from bovine liver, bovine serum albumin, 4-Amino-3-hydrazino-5-mercapto-1,2,4-triazole (Purpald), hexadecyltrimethylammonium bromide (HTAB), sodium deoxycholate, and Trizol were purchased from Sigma-Aldrich (Saint Louis, MO, USA). Xylazine hydrochloride and ketamine hydrochloride were obtained from Sespo Industry (Paulínea, SP, Brazil). Sodium chloride, hydrogen peroxide, sodium hydroxide, potassium hydroxide, and iodine periodate were obtained from Synth Chemistry (São Paulo, Brazil). 2-nitrobenzoic acid (DTNB), hypoxanthine, and nitroblue tetrazolium salt (NBT) were obtained from Alfa Aesar (Tewksbury, MA, USA). Methanol was purchased from JT Baker (Xalostoc, Mexico). Phenylmethanesulfonyl fluoride (PMSF) was purchased from Amresco (Solon, OH, USA). Biuret reagent was purchased from Laborclin (Belo Horizonte, MG, Brazil). Hematoxylin and Eosin (H&E), periodic acid-Schiff (PAS), xylol, liquid paraffin, and sodium dodecyl sulfate (SDS) were purchased from Merck (Darmstadt, Germany). Ethylenediamine tetraacetic acid (EDTA), phosphate-buffered saline (PBS), and protease inhibitor cocktail was purchased from Thermo Fisher Scientific (Waltham, MA, USA).

### Induction of ulcerative colitis

**Fig. 1:**
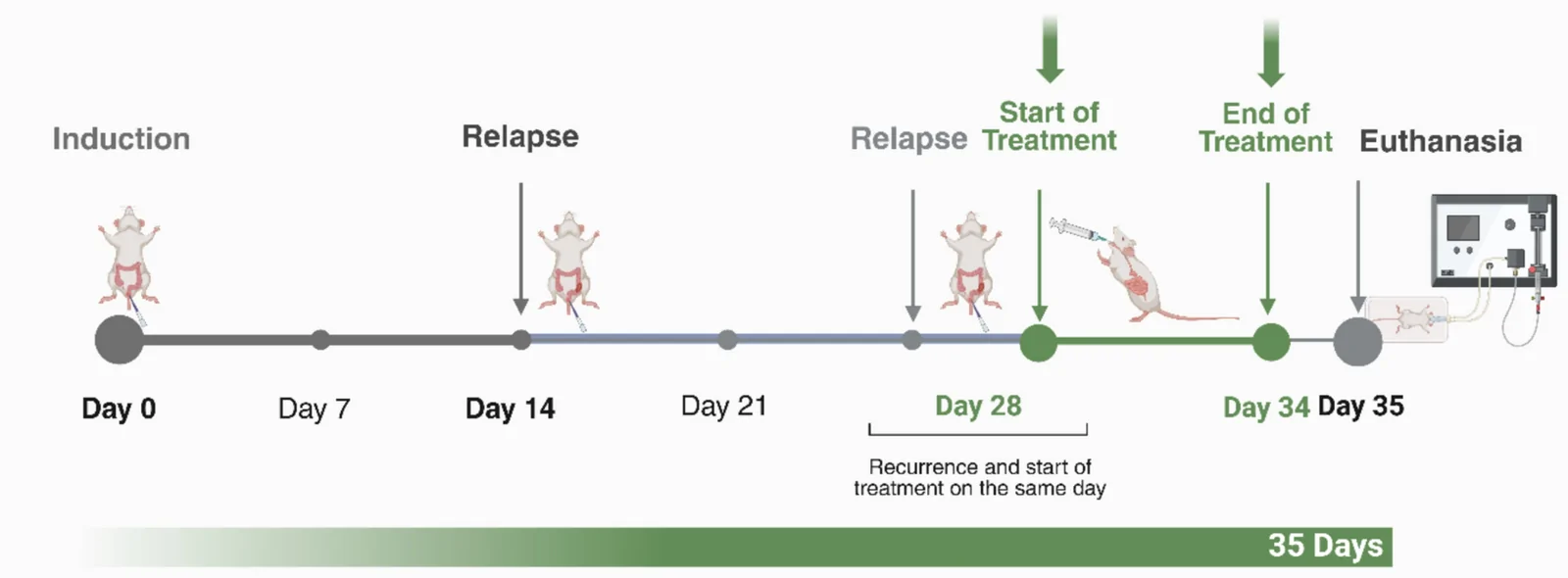
Schematic representation of the TNBS-induced relapsing colitis model in rats. TNBS was administered intrarectally on days 0, 14, and 28 to induce relapses. Following the final induction, the animals received oral treatment with EBi or the vehicle for seven days. Created in BioRender®

For the experimental protocol, all animals were anesthetized with xylazine and ketamine (15 and 100 mg/kg, respectively), followed by colitis induction with relapses by intracolonic administration of TNBS (2,4,6-trinitrobenzene sulfonic acid, 3 mg/kg dissolved in 50% ethanol) through a catheter on days 0, 14, and 28. The sham group was anesthetized but not treated with TNBS. On the twenty-eighth day, the animals received EBi (62.5, 125, and 250 mg/kg; doses were selected from previous studies with the extract, unpublished data) or vehicle (NaCl 0.9%, 10 mL/kg) orally by gavage for 7 days (Fig. 1). The EBI was prepared as previously described ^14^ using plant material collected and processed under SisGen authorization (Registration No. A29AEE3), in compliance with Brazilian genetic heritage legislation. On day 35, the rats were euthanized by anesthesia with isoflurane (induction: 2%, maintenance: 2.5–3%; Isoforine® Cristália–Produtos Químicos Farmacêuticos Ltda., Itapira, SP, Brazil) for subsequent cardiac puncture, and their colons were removed for macroscopic examination (Gotardo et al. 2014). The samples were then stored in a freezer at − 80 °C until biochemical analysis.

### Damage score

An independent researcher verified the severity of colitis using a previously reported score (Morris et al. 1989). The colon was opened longitudinally and cleaned with saline solutions to eliminate fecal residue. A trained observer analyzed the tissue based on the following criteria: 0 (no damage), 1 (presence of local hyperemia and no visible ulcer), 2 (ulceration without hyperemia or bowel wall thickening), 3 (ulceration with one focus of inflammation), 4 (presence of two or more sites of inflammation), and 5 (major sites of damage > 1 cm in colon length).

### Determination of malondialdehyde (MDA) levels

Lipid peroxidation was determined by measuring the reaction of thiobarbituric acid substances with colon samples. Test tubes were filled with 400 *µ*L of distilled water, 200 *µ*L of 8.1% lauryl sulfate, 1500 *µ*L of 20% acetic acid pH 3.5, 1500 *µ*L of 0.8% thiobarbituric acid (TBA) diluted in 20% acetic acid, and 400 *µ*L of sample for the colorimetric reaction. The concentration of thiobarbituric acid reactive substances was measured at 532 nm, and the results were expressed in nmols of MDA/g of tissue (Ohkawa et al. 1979).

### Myeloperoxidase (MPO) activity determination

MPO levels were determined using a previously established protocol (Krawisz et al. 1984). Briefly, 50 mg of colon tissue was homogenized in 1 mL of HTAB buffer with a Polytron Homogenizer (five cycles of maximum rotation) on ice (4 °C). The supernatant was separated by centrifugation (11,180 × g at 4 °C) for 10 min, and 50 *µ*L was collected for enzymatic analysis. The volume was combined with 200 µL of a solution containing ortho-dianisidine dihydrochloride in phosphate buffer (37 °C) and measured using a spectrophotometer at 450 nm. The results were expressed as U/g of tissue.

### Catalase (CAT) activity

Colon strips were homogenized 1:4 (w/v) in extraction buffer (1% Triton X-100, 100 mM Tris–HCl (pH 7.4), 100 mM sodium pyrophosphate, 100 mM sodium fluoride, 10 mM EDTA, 10 mM sodium orthovanadate, 2.0 mM PMSF and 0.1 aprotinin/mL) at 4 °C and centrifuged for 45 min at 21,900 g. The samples were diluted 1:20 (5 *µ*L of supernatant and 95 *µ*L of PBS) with 100 *µ*L of assay buffer (KH_2_PO_4_, 250 mM, pH 7), 30 *µ*L methanol, and 20 *µ*L of hydrogen peroxide (35.3 mM). Next, 30 *µ*L of hydrogen hydroxide (10 M) and 45 *µ*L of Purpald (34.2 mM) were added, followed by incubation for 10 min. Next, 15 *µ*L of potassium periodate (65.2 mM) was added and incubated for 5 min at room temperature in the dark. Absorbance was measured at 540 nm (Aebi 1984). The results are expressed in U/g of tissue.

### Superoxide dismutase (SOD) activity

The tissue was homogenized and diluted 1:10 (15 and 285 *µ*L of phosphate-buttered saline [PBS]). The samples were centrifuged at 11,180 × g at 4 °C for 15 min. Subsequently, 150 *µ*L of a solution containing hypoxanthine, xanthine oxidase, and nitroblue tetrazolium hydrochloride (1:1:1, v/v) was added to each sample, and the absorbance was measured for 10 min at intervals for 1 min. SOD activity was determined by dividing the AUC of the samples by the total amount of protein and multiplying it by the dilution factor (Winterbourn et al. 1975). The results are expressed in U/g of tissue.

### Reduced glutathione (GSH) levels

Colon samples were homogenized using an extraction buffer, diluted 1:20 (30 *µ*L of samples + 270 *µ*L PBS), and centrifuged at 11,180 × g at 4 °C for 15 min. The supernatant (100 *µ*L) was added to a 96-well plate containing Tris–EDTA buffer (1 mM/2 mM, pH 8.2). Subsequently, 100 *µ*L of the standard curve containing L-reduced Glutathione (0–500 nmol/mL) and 100 *µ*L of the sample were added, and the first read was performed at 412 nm. Following this step, 20 *µ*L of 5,5 dithiobis-2-nitrobenzoic acid (10 mM) solubilized in methanol was added, and the plate was protected from light. A second reading was obtained at the same wavelength after 15 min of incubation at room temperature. The results were expressed as nmol/g of tissue (Faure and Lafond 1995).

### Measurement of cytokines

Colon samples were homogenized in extraction buffer and centrifuged at 21,900 g for 45 min. The concentrations of TNF-*α* (SRTA00), IL-1*β* (SRLB00), and IL-10 (SR1000) were determined using immunoenzymatic assays with R&D kits, following the manufacturer’s instructions.

### Histological analysis

Colon specimens were placed in neutral tamponade formalin (10%) and immersed in formaldehyde for 72 h. The biopsies were identified and inserted into appropriate recipients. The tissue was processed using the following steps: water bath, dehydration with crescent alcohol concentration, diaphanization with xylol, and placement in a bath containing liquid paraffin at a mean temperature of 60 °C. The slices were then embedded in paraffin blocks and subjected to microtomy, with each specimen resulting in six longitudinal cuts with a thickness of 5 *µ*m. For H&E staining, the colon samples were subjected to two baths of xylol for 10 min each. Subsequently, the slices were subjected to three baths of absolute alcohol and were hydrated with water. The final step involved immersing the slices in Harris Hematoxylin for 1 min, washing them to remove excess tissue, and exposing them to eosin for an additional 2 min. This was followed by raising and dehydrating in three alcohol baths, a xylol/alcohol bath, and three xylol baths. For periodic acid-Schiff (PAS) staining, the slices were exposed to periodic acid for a minimum of 60 min at room temperature. Subsequently, the slides were subjected to a 3 min wash with water, followed by a 3-min wash with distilled water at room temperature. The material was then exposed to Schiff’s reagent for 60 min and washed with distilled water for 5 min. The slices were stained with Harris Hematoxylin for 3 min, dehydrated in three successive alcohol baths, and diaphanized in three xylol baths. Finally, the slices were mounted on coverslips and resins.

#### Quantitative real-time PCR

Colon samples were extracted using magnetic beads, PreCellys24 equipment (Montigny-le-Bretonneux, France), and TRIzol reagent, following the manufacturer’s protocols. Total RNA was diluted in 80 µL of nuclease-free water and stored at − 80 °C. RNA concentration was determined using a NanoDrop 2000 spectrophotometer. One microgram of contaminant-free RNA was converted to cDNA using the High-Capacity Reverse Transcriptase Kit (Thermo Fisher Scientific), according to the manufacturer’s instructions, and double-stranded DNA was measured using Nanodrop to confirm reaction efficiency. Primers (Table 1) were designed using Primer 3 (https://primer3.ut.ee/) and synthesized by Exxtend Biotechnology (Paulínea, SP, Brazil). Quantitative real-time polymerase chain reaction (qPCR) was performed using Go Taq qPCR Master Mix (Promega, Madison, WI, USA), and the selected housekeeping gene was glyceraldehyde-3-phosfate-dehydrogenase (GAPDH) was selected as the housekeeping gene. The expression levels were determined using the 2^−DDCT^ method (Livak and Schmittgen 2001; Untergasser et al. 2012).

**Table 1:** Genes selected to characterize the mechanism of action of *Byrsonima intermedia* in TNBS-induced IBD in rats

*Gene*	Sequência 5’-3’	GenBank
*GAPDH*	5′-GCCGAAGTACCCAAGGAGAC -3′	24,383
	5′-AAAGGCGGAGTTACAAGGGG-3′	
*CLDN-1*	5′- AGTTCCTTCCCTACCCTGGA-3′	65,129
	5′- TCCTCTGCCACACTCATCTG-3′	
*OCLN*	5′- CCAGTGTCTTGAGTGCCTGA-3′	83,497
	5′- TTCTGGCTTGTATGCCTGTG-3′	
*IL-1β*	5′- CTGTGACTCGTGGGATGATG-3′	24,494
	5′- AGACAGCACGAGGCATTTTT-3′	
*TNF*	5′-GGGAGACGAGGGAGATAAGG-3′	24,835
	5′- TTTATCCGTCCAACCTCAGC-3′	
*IL-6*	5′- CTGTTAGTCCCCATGCCAGT- 3′	24,498
	5′-GCATTCCTTCACCTTTTCCA -3′	
*IL-10*	5′-CTCCACCTGGCAAACAAAAT -3′	25,325
	5′-CTGCCTAGCCCACAAAGAAG -3′	
*NRF2*	5′-GCTCTTGGAGAGGACCTGTG -3′	83,619
	5′- CGGTCAGAAGAGGGTGTCAT-3′	
*MCP-1*	5′- TGTGCTCCCTGCTTCTCTTT-3′	24,770
	5′- CCGACTCATTGGGATCATCT-3′	
*ZO-1*	5′-CTGTGGGTTCCGTTTTGAGT -3′	292,994
	5′-CAGAAGGCCAAAGACTCCAG-3′	
*TLR4*	5′-AGAAAATGCCAGGATGATGC-3′	29,260
	5′-AGGGATTCAAGCTTCCTGGT-3′	

### Statistical analysis

The results are expressed as the mean ± standard error of the mean (S.E.M.). Statistical analyses were performed using the Kolmogorov–Smirnov normality test, and homoscedasticity was verified using Bartlett´s test. One-way analysis of variance (ANOVA) was used to compare three or more groups when the data were parametric. Non-parametric data were analyzed using the Kruskal–Wallis or Mann–Whitney tests. In the presence of significance, we proceeded with the analysis of contrast using the Dunnett or Dunn post-test, considering a significance level of 0.05, rejecting the null hypothesis, and confirming the alternative hypothesis.

## Results

### Macroscopic evaluation and MPO activity in colon tissue from colitis animals treated with EBi

**Fig. 2:**
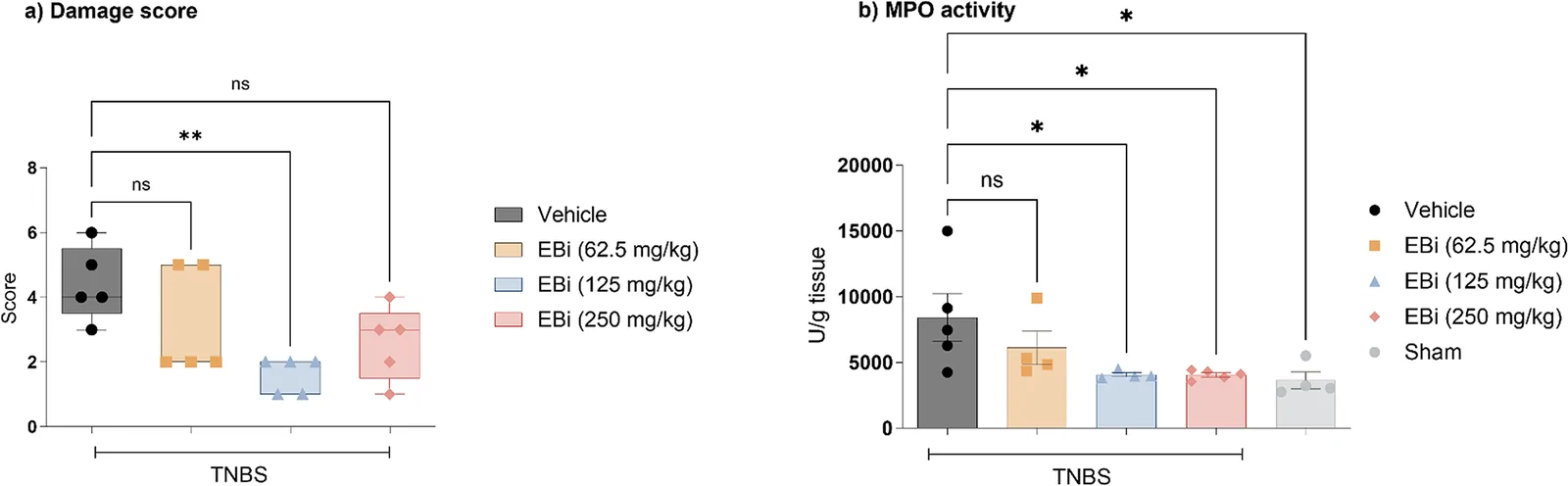
Effect of oral treatment with EBi (62.5, 125, or 250 mg/kg) in an experimental colitis model induced by repeated intracolonic TNBS administration. **a** Damage score. **b** MPO activity. Data are presented as mean ± S.E.M. (*n* = 4–5 per group). ANOVA followed by the Kruskal-Wally’s followed by the Dunn test for damage score, where ** *p* < 0.01 and ANOVA followed by Dunnett’s test, for MPO activity; * *p* < 0.05, compared to the respective vehicle group, ns = not significant

Repeated intracolonic TNBS administration in Wistar rats resulted in macroscopic lesions characterized by hyperemia and small ulcers in the colon (Fig. 2), which were macroscopically reduced by oral treatment with EBi (125 mg/kg) for seven days (Fig. 2a). Rats subjected to EBi treatment showed a lower impact on TNBS-induced colonic damage than those in the colitis group. Treatment with EBi (125 mg/kg) reduced the damage caused by TNBS instillation by 63% compared to that in the vehicle group (*p* < 0.05). Quantification of MPO activity demonstrated that treatment with EBi at doses of 125 and 250 mg/kg reduced neutrophilic infiltration compared to that in the vehicle group (Fig. 2b). The complete body-weight time course for all experimental groups is presented in *Supplementary Material (*Fig. S1*)*.

**Fig. 3:**
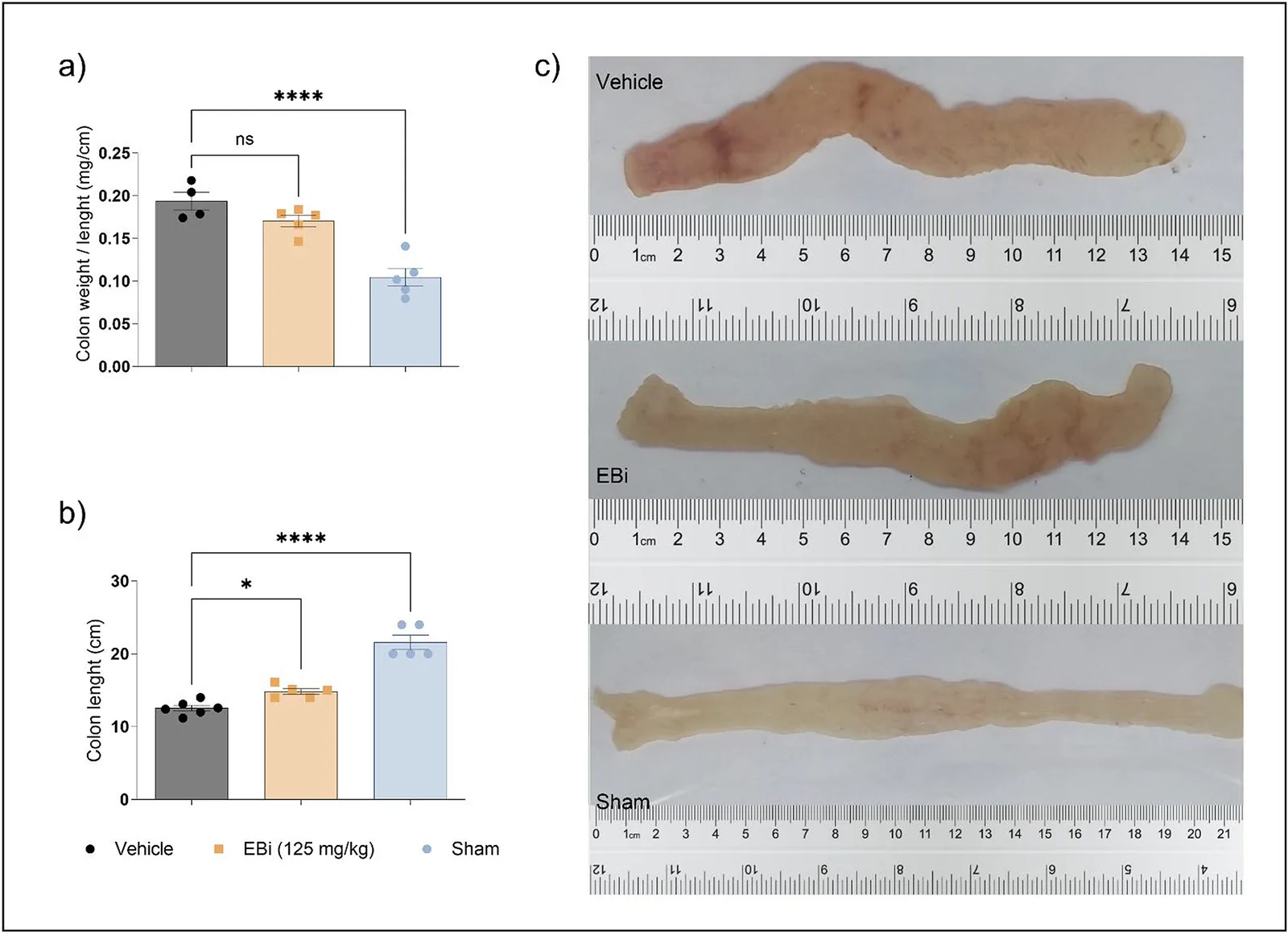
Oral treatment with EBi (125 mg/kg) reverted the TNBS-induced reduction in colon length. **a** Ratio of colon weight to length. **b** Colon weight. **c** Representative images of the colon of rats in each experimental group. Data are presented as mean ± S.E.M. (n = 5–6 per group). ANOVA, followed by Dunnett’s test. * *p* < 0.05 and **** *p* < 0.001 compared to the vehicle group

Colon weight and length-to-weight ratio remained unaffected by the treatment with extract (Fig. 3a). However, treatment with EBi (125 mg/kg) mitigated TNBS-induced colonic shortening compared to the vehicle group (Fig. 3b), as demonstrated by representative images of each experimental group.

### Analysis of antioxidant parameters

**Fig. 4:**
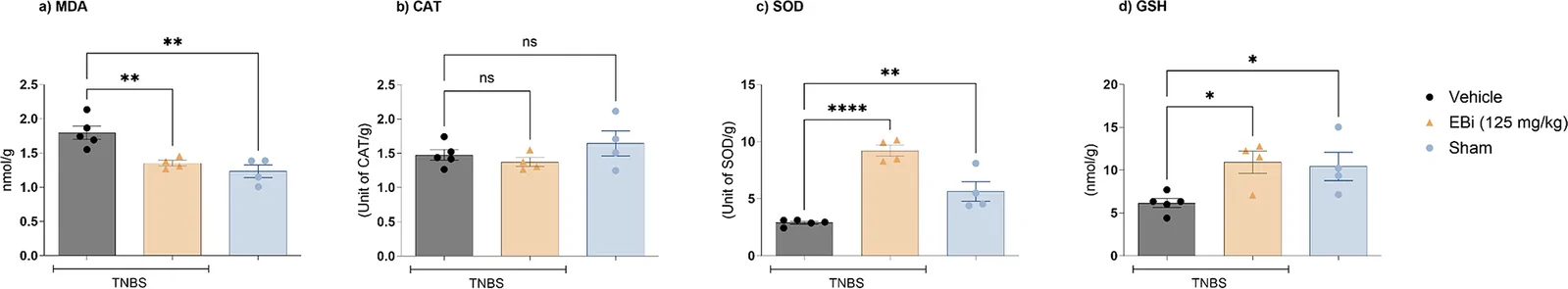
Antioxidant parameters in the colon of rats treated with EBi (125 mg/kg) after repeated TNBS administration. **a** MDA levels. **b** CAT activity, **c** SOD activity, and **d** reduced GSH levels. Data are presented as mean ± S.E.M. (n = 5–6 per group). ANOVA followed by Dunnett’s test, where * *p* < 0.05, **p < 0.01, *** *p* < 0.001 compared to the respective vehicle group

Colonic damage caused by intracolonic administration of TNBS increased MDA levels (Fig. 4a), decreased SOD activity (Fig. 4c), and reduced GSH levels (Fig. 4d) in the vehicle group compared to those in the sham group. Therefore, treatment with EBi (125 mg/kg) for 7 days significantly reduced MDA levels compared to the vehicle group (Fig. 4 a). This result was associated with a significant increase in SOD activity in the groups treated with EBi 125 mg/kg compared to that in the vehicle-treated group (Fig. 4c). The same doses of EBi effectively increased GSH levels incrementally compared to the vehicle group (Fig. 4d).

### Analysis of gene expression of factors related to IBD

**Fig. 5:**
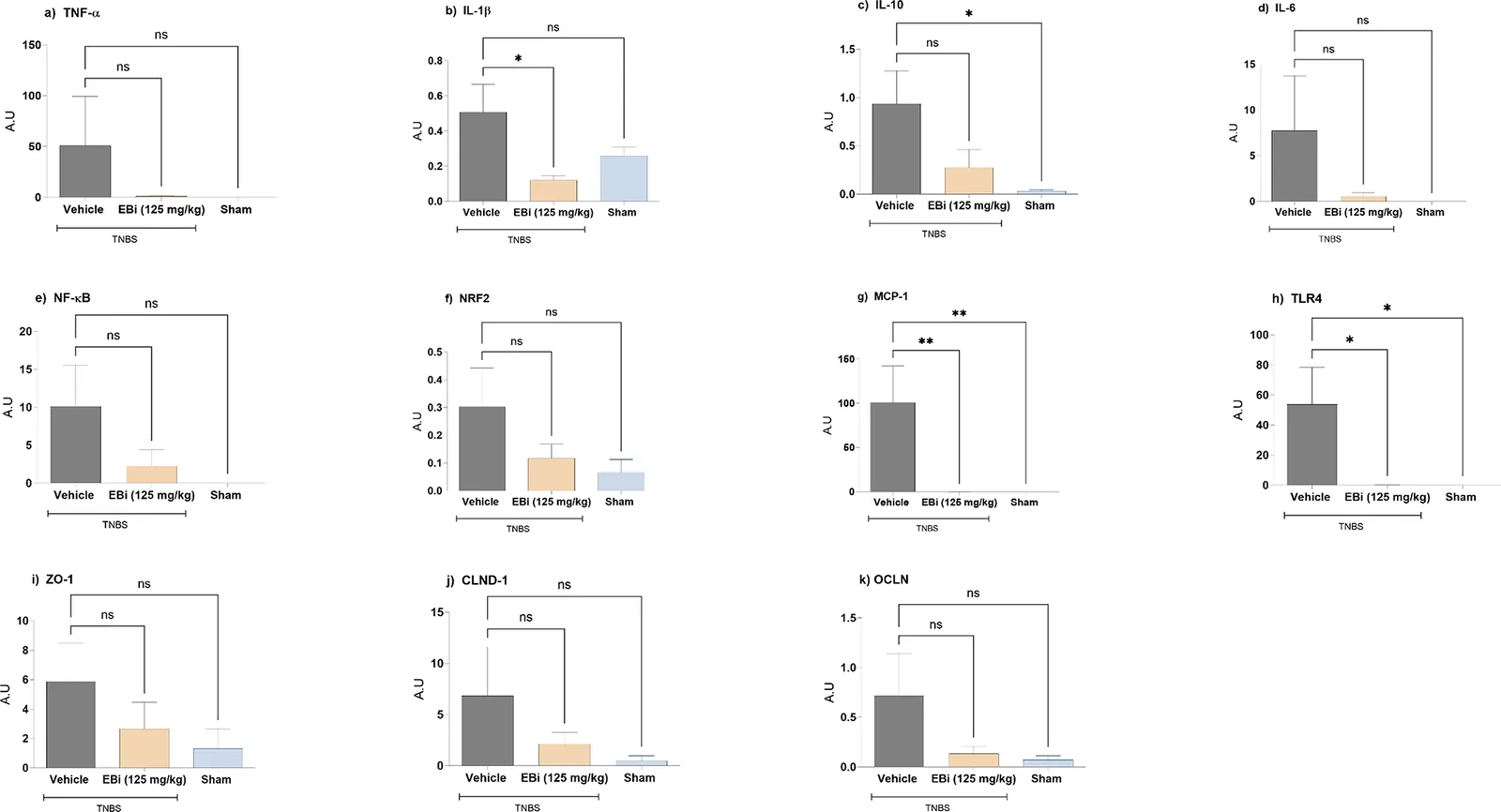
Effect of *Byrsonima intermedia* (EBi) on colitis induced by TNBS. Relative quantification of mRNA expression of **a** TNF-*α*, **b** IL-1*β*, **c** IL-10, **d** IL-6, **e** NF-κB, **f** NRF2, **g** MCP-1, **h** TLR-4, **i** ZO-1, **j** CLND-1 and **k** OCLN after 7 days of treatment. The bar represents the mean ± S.E.M. (n = 4–5) Statistical significance was determined using ANOVA followed by Dunnett’s test; * *p* < *0.05* and ** *p* < *0.01* compared with the vehicle

TBNS administration resulted in an inflammatory process with intestinal damage in rats (Fig. 5), which was accompanied by an increase in the gene expression of MCP-1 and TLR-4 in colonic tissue when compared between the vehicle and sham groups (Fig. 5g and h). Treatment with EBi (125 mg/kg) for 7 days significantly reduced the expression of *Il-1β*, *mcp-1*, and *tlr4* (Fig. 5b, g, and h) compared to that in the vehicle group. *IL-1β* expression was also significantly reduced after EBi treatment (Fig. 5b).

### Analysis of anti-inflammatory activities

**Fig. 6:**
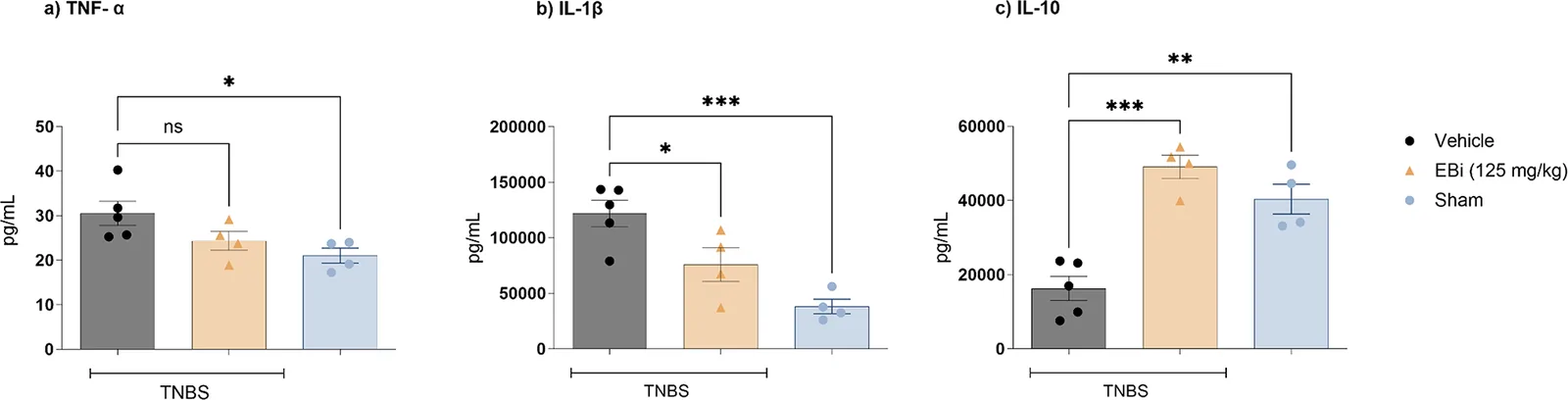
Assessment of TNF-*α* (**a**), IL-1*β* (**b**), and IL-10 (**c**) levels in the colon of rats after treatment with EBi in the TNBS-induced ulcerative colitis model. Data are presented as mean ± S.E.M. (n = 4–5 per group). ANOVA followed by Dunnett’s test, where * *p* < 0.05 and ** *p* < 0.01, *** *p* < 0.001 compared to the respective vehicle group

The levels of TNF-*α*, IL-1*β*, and IL-10 in the colon were also associated with inflammation in TNBS-induced colitis (Fig. 6). Intestinal inflammation caused a significant increase in TNF-*α* (Fig. 6a) and IL-1*β* levels (Fig. 6b), associated with a decrease in IL-10 levels (Fig. 6c) in the vehicle group compared to that in the sham group. However, treatment with EBi (125 mg/kg) resulted in significant decrease in IL-1*β* levels compared to those in the vehicle-treated group (Fig. 6b). Furthermore, it significantly increased in IL-10 levels in the colon after TNBS-induced intestinal inflammation compared to that in the vehicle group (Fig. 6c).

### Histological analyses

**Fig. 7:**
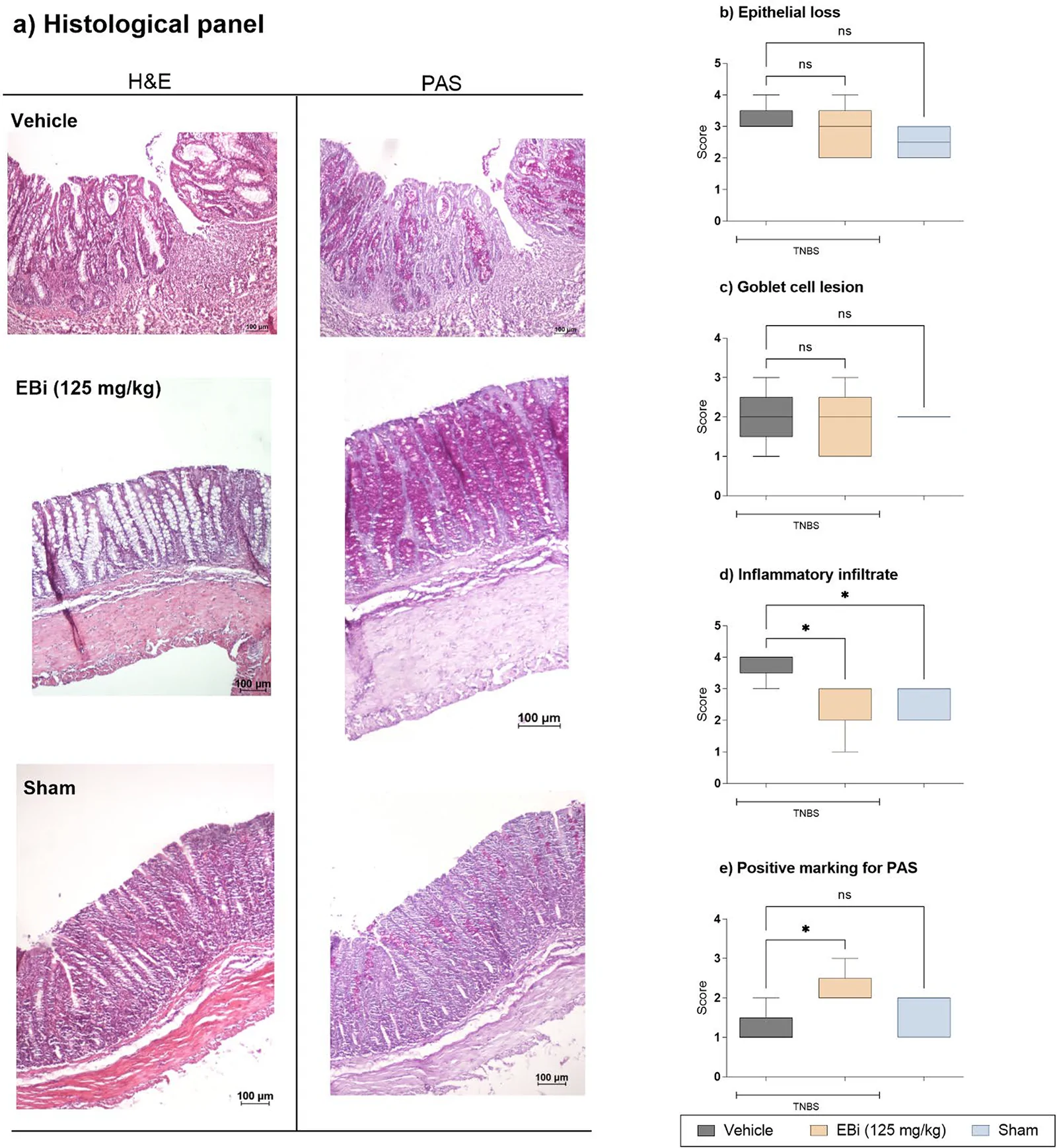
Histological analysis of rat colons after TNBS-induced intestinal inflammation. **a** The images represent each experimental group. H&E staining is shown on the left, and PAS staining is shown on the right. **b** The analysis of epithelial loss. **c** Goblet cells. **d** Inflammatory infiltrate. **e** Positive marking for PAS. Mean ± S.E.M. (n = 4–5 per group). Statistical significance was determined using ANOVA followed by Dunn’s test; * *p* < 0.05 compared with the vehicle

Histological examination of colonic tissues from animals subjected to TNBS-induced colitis revealed a significant reduction in inflammation following oral administration of EBI, as evidenced by a decrease in inflammatory cell infiltration in the histological sections of the colon (Fig. 7d).

## Discussion

In this study we demonstrated the protective and healing effects of EBi treatment on recurrent TNBS-induced intestinal inflammation in rats. These effects were mediated by a reduction in colonic damage and inflammation associated with lesion progression. The minimum dose of EBi (125 mg/kg) increased antioxidant capacity through SOD activity and GSH levels, alleviating lipid peroxidation in the colons of rats. In addition, the same doses exhibited an anti-inflammatory role, with a reduction in the pro-inflammatory mediator IL-1*β* and an increase in the levels of IL-10. This is the first study to demonstrate that the therapeutic effect of EBi against colitis is mainly due to the anti-inflammatory and antioxidant roles of the extract, which showed an improvement in colonic injury and epithelial integrity, as demonstrated by histological analyses.

The pharmacological potential of EBi has been demonstrated in studies involving gastrointestinal diseases, such as peptic ulcers and intestinal ischemia/reperfusion. These mechanisms are related to increased glutathione reductase content, nitric oxide levels, and the activation of neuronal capsaicin-sensitive cells (Santos et al. 2012; de Cassia Dos Santos et al.. 2019). However, the effects of colitis on relapse models have not yet been investigated. To achieve this, we established an experimental colitis model by intrarectal administration of TNBS. This model was created by Morris (Morris et al. 1989) and adapted by Gotardo (Gotardo et al. 2014) to explore the cycles of remission and relapse observed during the natural history of IBD. Our first result showed that oral treatment with 125 mg/kg reduced the damage score compared to that in vehicle-treated animals. This reduction was accompanied by a decrease in MPO activity in animals treated with 125 and 250 mg/kg EBi. MPO is a neutrophilic enzyme that acts as a biomarker of inflammation, indicating the infiltration of inflamed tissue (Yoshida et al. 1995). When the gut barrier is disrupted, neutrophils are the first cells recruited by intestinal epithelial cells via cytokines, chemokines, lipid mediators, and other molecules. Neutrophil migration is associated with disease symptoms and breakdown of cell junctions, leading to crypt disorganization and abscess formation (Danne 2024). At doses of 125 and 250 mg/kg, EBi significantly decreased MPO activity compared with control, indicating a reduction in neutrophil activity, possibly by regulating the inflammatory environment and reducing chemokine production.

This inflammatory process caused by TNBS administration resulted in an increase in MDA formation in vehicle-treated animals compared to that in sham animals. EBi oral treatment markedly reduced lipid peroxidation at a dose of 125 mg/kg compared to that in vehicle-treated animals, restoring MDA levels to those observed in sham animals. MDA is the final product of lipid peroxidation and is formed when reactive oxygen species (ROS) attack polyunsaturated fatty acids, notably linoleic acid, which is present in phospholipid membranes (Lei et al. 2021). This marker is elevated in dextran sulfate sodium (DSS) and TNBS colitis models (Muthuraman and Singh 2011; Tao et al. 2023; Bi et al. 2024). Treatment with 125 mg/kg EBi increased SOD activity and reduced GSH levels compared to vehicle-treated animals, indicating potent antioxidant capacity. These two enzymes are components of the redox system and show reduced activity in animals subjected to TNBS administration (Pavan et al. 2021; Arunachalam et al. 2021; Rong et al. 2021; Ye and Lai 2021). SOD is involved in to the conversion of the reactive anion superoxide into a less reactive species, hydrogen peroxide (Liu et al. 2022). Recent studies have shown that liposomal SOD administration can alleviate DSS-induced experimental colitis and reduce disease signs, such as colon shortening and diarrhea (Zhang et al. 2023), indicating the relevance of the SOD pathway in colitis development. *SOD2* expression is affected by colitis and is reduced in patients with CD. Simultaneously, *SOD1* levels are elevated, demonstrating that the SOD pathway is dysregulated during CD (Tavassolifar et al. 2021). GSH is a non-enzymatic antioxidant in the cytosol and nucleus that neutralizes oxidative agents through its thiol group, thereby limiting the extent of oxidative signaling (Diaz-Vivancos et al. 2015). Evidence shows that reduced expression of glutathione-related enzymes, such as GPX4, is correlated with oxidative damage and bacterial invasion in the ileum (Wen et al. 2023).

EBi treatment reduced the expression of genes associated with inflammatory processes, such as *MCP-1*, *TLR4 (Toll-like receptor 4)*, and *IL-1β* in the rat gut. MCP-1 is expressed by endothelial cells, monocytes/macrophages, and fibroblasts, and drives leukocyte migration into various disorders, such as IBD (Singh et al. 2021). TLR4 is a receptor expressed in immune cells that senses the presence of lipopolysaccharide (LPS) and triggers pro-inflammatory cytokine production (Zhang et al. 2022a). EBi at 125 mg/kg completely reduced the expression of *MCP-1* and *TLR4* to levels observed in sham animals, indicating that the LPS/TLR4/IL-1*β* pathway was inhibited after EBi treatment. The presence of microorganisms and colon damage triggers an innate and adaptive response, in which cells such as neutrophils and macrophages are recruited to the site of inflammation and release cytokines such as TNF-*α*, IL-1*β*, and IL-6 (Saez et al. 2023). In contrast, cytokines associated with anti-inflammatory action, such as IL-10, show reduced expression (Wu et al. 2023). EBi did not alter the protein content of TNF-*α* compared to that in the vehicle-treated animals. In contrast, EBi treatment for seven days promoted a significantly reduced in IL-1*β* levels and elevated IL-10 levels, suggesting that an anti-inflammatory response is stimulated by oral treatment with the extract. IL-10 regulates exuberant immune responses and prevents cytokine and chemokine release. This cytokine is produced by various immune cells, including T cells, B cells, natural killer (NK) cells, dendritic cells, macrophages, mast cells, and neutrophils (Wei et al. 2020).

TNBS-induced lesions result in significant alterations to colonic architecture, characterized by intestinal inflammatory infiltrates, edema, hyperemia, ulceration, epithelial cell loss, crypt distortion, and colonic wall thickening (Horn et al. 2023; Katsandegwaza et al. 2022; Bilsborough et al. 2021). These morphological changes were confirmed by histological analysis. In the present study, EBi treatment mitigated inflammatory infiltration and promoted the restoration of intestinal villi. H&E staining revealed more necrotic and erosive regions in the vehicle group than in the EBi-treated or sham groups, with no differences in epithelial loss and goblet cell injury. The pronounced inflammatory infiltrate observed in the vehicle-treated rats was significantly mitigated by EBi treatment. These findings reinforce the anti-inflammatory potential of EBi and its ability to facilitate colonic tissue regeneration in a rat model of IBD relapse. Together, these results demonstrate the protective effect of EBi on intestinal morphology in TNBS-induced colitis.

Quercetin was an important flavonoid in these extracts. Lyu et al. (Lyu et al. 2022) discussed the therapeutic potential of this molecule through mechanisms similar to those found in this study, whereas Zhang et al. (Zhang et al. 2022b) demonstrated how this flavonoid reduces inflammatory processes. Although it is not possible to identify the compound responsible for the activity of EBi, it is possible to state that it is a promising species against several disorders that affect the gastrointestinal tract (Moreira et al. 2011; Orlandi et al. 2011; Santos et al. 2012; Pereira et al. 2015). Further studies are necessary to characterize the effect of each compound on the pharmacological activity of the extract against TNBS-induced colitis in rodent models. Furthermore, it is crucial to evaluate the effects of EBi on other colitis models and the regenerative processes in intestinal lesions. In this study, we demonstrated the pharmacological effect of EBi against the intestinal inflammatory process induced by TNBS only in male rats. Despite the therapeutic potential of the extract, sex hormones (such as estrogen and progesterone) are known to influence the development and progression of IBD (Pan et al. 2024). Therefore, future studies may be characterizing the effects of different doses and evaluate the activity of EBi in female animals. In addition, it would be relevant to assess the disease activity index and analyze the intestinal microbiota to establish correlations with clinical parameters, which may further elucidate the mechanisms underlying the effects of the extract and contribute to a more translational approach. eThese findings will help bring the extract closer to clinical application as a potential therapeutic alternative for IBD.

## Conclusion

This study presents evidence that EBi treatment alleviates the severity of TNBS-induced intestinal inflammation by promoting reparative effects on the gut. These effects are partly attributed to a reduction in the damage score and macroscopic lesions during colitis, a decrease in MPO activity and lipid peroxidation, and an enhancement of antioxidant defenses (SOD and GSH). Furthermore, there was a notable upregulation of IL-10, accompanied by a downregulation of IL-1*β* and TLR4. Histological analysis confirmed the improvement in the inflammatory process, supporting previous studies that have suggested the use of *Byrsonima intermedia* as a therapeutic agent for gastrointestinal diseases.

## Supplementary Information

Below is the link to the electronic supplementary material.Supplementary file1 (DOCX 102 kb)

## Data Availability

No datasets were generated or analysed during the current study.
