# Supplementary material for: The methanolic extract of Byrsonima intermedia mitigates colonic injury in experimental colitis through redox restoration, cytokine modulation, and tissue repair
Source: Inflammopharmacology. 2026 Jul 14;34(8):5619–31. doi: 10.1007/s10787-026-02312-4 (PMC13525043; doi:10.1007/s10787-026-02312-4)
Supplement: Supplementary file 1 [file 10787_2026_2312_MOESM1_ESM.docx]

**Supplementary Figure S1.** *Body‑weight trajectory of rats during the TNBS‑induced relapse colitis model and subsequent treatment with Vehicle or EBi (62.5, 125, or 250 mg/kg). Measurements were recorded throughout the experimental timeline. Data are presented as mean ± SEM (n = 5/group). Statistical analysis was conducted using two‑way ANOVA followed by Dunnett’s post hoc test to compare treatment groups with the Vehicle control.*
